# Disrupted resting-state functional architecture of the brain after 45-day simulated microgravity

**DOI:** 10.3389/fnbeh.2014.00200

**Published:** 2014-06-05

**Authors:** Yuan Zhou, Yun Wang, Li-Lin Rao, Zhu-Yuan Liang, Xiao-Ping Chen, Dang Zheng, Cheng Tan, Zhi-Qiang Tian, Chun-Hui Wang, Yan-Qiang Bai, Shan-Guang Chen, Shu Li

**Affiliations:** ^1^Key Laboratory of Behavioral Science and Magnetic Resonance Imaging Research Center, Institute of Psychology, Chinese Academy of SciencesBeijing, China; ^2^University of Chinese Academy of SciencesBeijing, China; ^3^National Key Laboratory of Human Factors Engineering, China Astronaut Research and Training CenterBeijing, China

**Keywords:** anterior insula, cingulate cortex, head-down tilt bed rest, functional magnetic resonance imaging (fMRI), functional connectivity, resting state

## Abstract

Long-term spaceflight induces both physiological and psychological changes in astronauts. To understand the neural mechanisms underlying these physiological and psychological changes, it is critical to investigate the effects of microgravity on the functional architecture of the brain. In this study, we used resting-state functional MRI (rs-fMRI) to study whether the functional architecture of the brain is altered after 45 days of −6° head-down tilt (HDT) bed rest, which is a reliable model for the simulation of microgravity. Sixteen healthy male volunteers underwent rs-fMRI scans before and after 45 days of −6° HDT bed rest. Specifically, we used a commonly employed graph-based measure of network organization, i.e., degree centrality (DC), to perform a full-brain exploration of the regions that were influenced by simulated microgravity. We subsequently examined the functional connectivities of these regions using a seed-based resting-state functional connectivity (RSFC) analysis. We found decreased DC in two regions, the left anterior insula (aINS) and the anterior part of the middle cingulate cortex (MCC; also called the dorsal anterior cingulate cortex in many studies), in the male volunteers after 45 days of −6° HDT bed rest. Furthermore, seed-based RSFC analyses revealed that a functional network anchored in the aINS and MCC was particularly influenced by simulated microgravity. These results provide evidence that simulated microgravity alters the resting-state functional architecture of the brains of males and suggest that the processing of salience information, which is primarily subserved by the aINS–MCC functional network, is particularly influenced by spaceflight. The current findings provide a new perspective for understanding the relationships between microgravity, cognitive function, autonomic neural function, and central neural activity.

## Introduction

With the launch of the first manned spaceflight by the Soviet Union on the 12th of April 1961 with cosmonaut Yuri Gagarin aboard, it became possible to travel beyond Earth's gravity. The success of interplanetary spaceflight will depend upon whether humans can function normally within an environment of microgravity and confinement (Basner et al., 2013). Physiological and psychological changes induced by long-term spaceflight have been observed in astronauts and cosmonauts (Williams et al., 2009). To understand the neural mechanisms underlying these physiological and psychological changes, it is critical to investigate the effects of microgravity on brain activity from the brain network perspective because our understanding of the functioning of the human brain ultimately depends upon our knowledge of large-scale brain organization (Bressler and Menon, 2010).

The intrinsic activity of the brain provides an important profile of brain function (Raichle, 2010b). The majority of the energy (60–80%) consumed by the brain is used to support intrinsic and spontaneous neural activity during rest (Raichle, 2010a). In normal-weight environments, resting-state functional magnetic resonance imaging (rs-fMRI) measurements of this intrinsic and spontaneous brain activity effectively reflect the brain's functional architecture while responding to external stimuli (Raichle and Mintun, 2006; Smith et al., 2009), as evidenced by findings that resting-state brain activity can predict the brain activities evoked by a variety of cognitive tasks (Fox et al., 2006, 2007; Mennes et al., 2010, 2011; Liu et al., 2011; Zou et al., 2012). Therefore, we hypothesize that studies of the effects of microgravity on the intrinsic functional architecture of the brain may provide valuable insight into the neural mechanisms underlying the physiological and psychological changes that occur in microgravity.

Although it is difficult to recreate the state of microgravity experienced by astronauts during stays in space on Earth, head-down tilt (HDT) bed rest (i.e., prolonged periods of rest in the HDT position) has proven to be a useful and reliable model for the simulation of most of the physiological (Pavy-Le Traon et al., 2007; Nicolas and Weiss, 2009; Moore et al., 2010) and psychological effects of spaceflight (De La Torre et al., 2012). Recently, the link between simulated microgravity and central neural activity has received increasing attention (Cheron et al., 2006; Schneider et al., 2008; Liao et al., 2012, 2013). A resting-state electroencephalograph study provided evidence that the blood oxygen level-dependent (BOLD) signal may be influenced by simulated microgravity (Cheron et al., 2006). The authors of this study found increased spontaneous alpha rhythm oscillation in response to simulated microgravity (Cheron et al., 2006), and this oscillation is inversely related to BOLD signal changes (Feige et al., 2005). Two recent resting-state BOLD fMRI studies provided evidence that local spontaneous brain activity is altered after short-term (72 h) −6° HDT bed rest (Liao et al., 2012, 2013). Although these studies suggest that the investigation of the effects of simulated microgravity on resting-state functional architecture using HDT bed rest is feasible, two questions remain unresolved. First, the effects of simulated microgravity on the functional networks of the brain have not yet been addressed. Second, the effects of prolonged HDT bed rest on the functional activity of the brain are unknown. Considering the different physiological effects of short-term and prolonged HDT bed rest that have been observed (Mano et al., 1998; Arbeille et al., 2001; Dyckman et al., 2012), it is necessary to investigate the effects of prolonged HDT bed rest on the functional activity of the brain.

In this study, we performed a full-brain exploration of the functional networks of the brain using rs-fMRI data obtained before and after 45 days of −6° HDT bed rest. To achieve this goal, we first analyzed voxel-wise network centrality. This graph-based measure of network organization accounts for the functional relationships of a given voxel (node) within the entire connectivity matrix of the brain rather than accounting for the relationships with specific nodes or networks (Buckner et al., 2009; Tomasi and Volkow, 2011; Zuo et al., 2012). Specifically, we used the commonly employed measure of degree centrality (DC) (Buckner et al., 2009; Zuo et al., 2012), which measures the number of direct connections of a given node. A node will have a high DC if it has numerous direct connections to other nodes. This measure has been widely used to detect changes in resting-state functional networks (Buckner et al., 2009; Fransson et al., 2011; Lord et al., 2011; Di Martino et al., 2013). Thus, the examination of voxel-wise DC allowed us to identify the brain regions that may be influenced by weightlessness without requiring a priori selection of nodes or networks of interest. Next, a seed-based resting-state functional connectivity (RSFC) analysis was used to further reveal the details of the functional networks that were associated with the identified regions (Buckner et al., 2009).

## Materials and methods

### Participants

Male volunteers were invited to participate in this 45 days of −6° HDT bed rest experiment. The volunteers were screened in two stages: first, the volunteers were interviewed about past and present physical and psychosocial statuses; second, the volunteers participated in a physical examination that included routine medical and laboratory tests to exclude chronic diseases. Sixteen volunteers between 20 and 32 years old (mean = 26.6, *SD* = 4.2) who were free of family histories of chronic medical or psychiatric diseases and free of neurological disorders, musculoskeletal system disorders, infectious diseases and dyssomnia, were not taking medications or drugs and had received greater than high school levels of education were selected. These 16 volunteers were paid for their participation. One participant was excluded from the fMRI analyses due to excessive head motion (please see the Data Preprocessing section).

A detailed explanation of the study (purpose and research hypotheses, experimental procedures and methods, research conditions, possible problems and complications) was provided, and written informed consent was subsequently obtained from each participant. Participants received financial compensation at the end of the study. The study was approved by the Institutional Review Board of the Institute of Psychology of the Chinese Academy of Sciences and the Institutional Review Board of the China Astronaut Research and Training Center.

### Bed rest procedure and design

The 16 volunteers each successfully completed 45 days of −6° HDT bed rest, which was implemented in three phases. In brief, the bed rest procedure included a 10-day baseline control period prior to the HDT bed rest (pre-HDT), a 45-day HDT bed rest period and a 10–15-day ambulatory recovery period after the HDT bed rest (post-HDT). During the HDT bed rest period, the volunteers were housed in three rooms and were under constant video surveillance. The participants performed all daily activities (including eating meals, washing, bathing, and urinating) lying down on their beds during the HDT bed rest period. The bedrooms were air-conditioned, and the room temperature was maintained between 23 and 27°C. Nursing care was provided throughout the duration of the study. Physicians examined the physical conditions of the participants regularly. The participants were allowed to freely communicate with each other and were free to engage in leisure activities, such as watching television and videos, listening to the radio and tapes, reading books and magazines, and making telephone calls.

To examine the differences in the resting-state functional networks between the normal and simulated microgravity conditions, each volunteer underwent two fMRI sessions; the first occurred on the 8th day of the pre-HDT period (normal gravity condition), and the second occurred on the 3rd day of the post-HDT period (simulated microgravity condition).

To detect whether there are changes of emotional state or not, we also used a Positive and Negative Affect Schedule (PANAS) to measure participants' emotions before entering the scanning room for each fMRI session. The Chinese version of the PANAS has well-established validation and reliability (Zhang et al., 2004). The schedule consists of 60 words and phrases that are ranked on a Likert scale from 1 (very much) to 5 (not at all). Participants were required to give the value according to their emotional state in the recent 1 week. The PANAS provides scores for the two primary factors, i.e., positive affect and negative affect. Lower scores for each factor indicate greater positive affect or negative affect, respectively.

### MRI data acquisition

The MRI data were acquired with a 3.0-Tesla Siemens MRI scanner in the BNU Imaging Center for Brain Research of the National Key Laboratory of Cognitive Neuroscience and Learning. Whole-brain functional scans were collected in 33 axial slices using an echo-planar imaging (EPI) sequence (repetition time = 2000 ms, echo time = 30 ms; flip angle = 90°, matrix = 64 × 64; field of view = 200 ×200mm^2^; slice thickness = 3.5mm; slice gap = 0.7mm). Each brain volume comprised 33 axial sections, and each functional run contained 200 volumes. High-resolution T1-weighted images were acquired in a sagittal orientation using an MPRAGE sequence (repetition/echo time = 2530/3.39 ms; flip angle = 7°; 1 × 1mm in-plane resolution; slice thickness = 1.33mm, no gap; 144 slices). After the collection of the resting-state fMRI data, several cognitive tasks were performed in the scanner. The fMRI data acquired during the execution of these tasks were not used in this study.

### Data preprocessing

Unless otherwise stated, all preprocessing was performed using the Data Processing Assistant for Resting-State fMRI (DPARSF 2.3, http://www.restfmri.net) (Yan and Zang, 2010), which is based on the Statistical Parametric Mapping (SPM8) program (http://www.fil.ion.ucl.ac.uk/spm) and the Resting-State fMRI Data Analysis Toolkit (REST 1.8, http://www.restfmri.net) (Song et al., 2011). Prior to preprocessing, the first 10 volumes were discarded to allow for signal stabilization. The remaining volumes acquired from each subject were corrected for the differences in slice acquisition times. The resultant images were then realigned to correct for small movements that occurred between scans. Based on the recorded motion correction estimates, the subjects with more than 2mm maximum displacement in any of the x, y, or z directions or more than 2° of angular rotation about any axis for any of the 190 volumes were excluded from this study. Based on this criteria, one volunteer was excluded from the analyses. Individual T1-weighted structural images were co-registered to the mean of the realigned EPI images. The transformed structural images were then segmented into gray matter, white matter, and cerebrospinal fluid (Ashburner and Friston, 2005). The Diffeomorphic Anatomical Registration Through Exponentiated Lie algebra (DARTEL) tool (Ashburner, 2007) was used to compute the transformations from individual native space to MNI space and vice-versa.

As resting-state MRI measures have been shown to be sensitive to micro-head motions (Yan et al., 2013a), the Friston 24-parameter model (Friston et al., 1996) was used to regress head motion effects out of the realigned data (the 24 parameters include 6 head motion parameters, 6 head motion parameters one time point before, and the 12 corresponding squared items) based on recent reports that have demonstrated that higher-order models benefit from the removal of head motion effects (Satterthwaite et al., 2013; Yan et al., 2013a). We further characterized the mean frame-wise displacement (FD), which considers measures of voxel-wise differences in motion in its derivation (Jenkinson et al., 2002), as a measure of the micro-head motion of each subject (Yan et al., 2013b).

To further reduce the effects of confounding factors, the signals from the white matter and cerebrospinal fluid, the mean time series of all voxels across the whole brain and linear and quadratic trends were removed from the data with linear regression (Yan et al., 2013b). Temporal filtering (0.01–0.1 Hz) of the time series was then performed.

### Degree centrality

To exclude artifactual correlations from non-gray matter voxels, we restricted our voxel-wise centrality analyses to a predefined gray matter mask that included tissue with gray matter probabilities greater than 20% as previously described (Zuo et al., 2012). This gray matter tissue probability template has been released as a part of the tissue priors in SPM8 (http://www.fil.ion.ucl.ac.uk/spm/software/spm8). The gray matter mask was warped into individual space using DARTEL information.

Within the study mask, individual network centrality maps were generated in a voxel-wise fashion. First, the preprocessed functional runs were subjected to voxel-based whole-brain correlation analysis. The time course of each voxel from each participant that was within the gray matter mask was correlated with the time course of every other voxel, which resulted in a correlation matrix. An undirected adjacency matrix was then obtained by thresholding each correlation at *r* > 0.25 (Buckner et al., 2009; Zuo et al., 2012; Yan et al., 2013a,b). Then, the DC was computed as the number of significant correlations (binarized) or as the sum of the weights of the significant connections (weighted) for each voxel (Zuo et al., 2012). Finally, the individual-level voxel-wise DC was converted into a z-score map by subtracting the mean DC across the entire brain and dividing by the standard deviation of the whole-brain DC (Zuo et al., 2012; Yan et al., 2013b). The resulting maps were then registered into MNI space with 3mm^3^ cubic voxels using the transformation information acquired from DARTEL. A smoothing kernel of 6mm was applied after registration.

Paired *t*-tests were performed to examine the differences between the DC measures before and after HDT bed-rest while accounting for the confounding effects of Jenkinson's mean FD by including this term as a regressor as recommended in a previous study (Yan et al., 2013a). Statistical significance was set at a voxel-wise *p* < 0.005 in conjunction with clusterwise FDR *p* < 0.05 to correct for multiple comparisons.

### RSFC networks

To reveal the specific networks that were influenced by HDT bed-rest, the regions selected based on the results of the DC analyses were used as seed regions for seed-based RSFC analyses. The mean time series of each seed region was acquired by averaging the time series of all of the voxels within that region. Pearson's correlation coefficients were then computed between the mean time series of the seed region and the time series of each voxel in the study mask. The correlation coefficients were then converted into *z*-values using Fisher's r-to-z transformation to improve their normality. Then, the individual *z*-values were entered into random effects one-sample *t*-tests in a voxel-wise manner to identify the brain regions that exhibited significant positive or negative correlations with the seed region within each condition (voxel-wise *p* < 0.01, clusterwise FDR *p* < 0.05). Next, a mask was generated by combining the regions that exhibited significant positive or negative connectivities with the seed region within each condition. Finally, the *z*-values were entered into paired two-sample *t*-tests in a voxel-wise manner to identify the brain regions that exhibited significant differences in positive or negative connectivity with the seed region between the pre-HDT and post-HDT conditions. This analysis was performed using the abovementioned mask to separate the positive and negative connectivities. Statistical significance was determined with a voxel-wise *p* < 0.005 in conjunction with a clusterwise FDR *p* < 0.05 to correct for multiple comparisons. In these group analyses, Jenkinson's mean FD was included as a nuisance regressor (Yan et al., 2013a).

### Validation analyses

To validate that our findings will not be due to confounding factors related to scanning interval, we repeated the principle analyses with an independent dataset (validation dataset). In brief, the dataset consists of 8-min resting-state fMRI scans that were acquired from 14 healthy adults without being exposed to the bed rest procedure at two different time points on a 3.0 T scanner (GE, Signa HDx, Renmin Hospital of Wuhan University). The mean interval duration was 46 days (46.4 ± 3.1). The scanning parameters were as follows. Whole-brain functional scans were collected in 32 axial slices using an EPI sequence (repetition time = 2000 ms, echo time = 30 ms; flip angle = 90°, matrix = 64 × 64; field of view = 220 × 220mm^2^; slice thickness = 4mm; slice gap = 0.6mm). High-resolution T1-weighted images were acquired in a sagittal orientation using a 3D BRAVO sequence (repetition/echo time = 7.788/2.984 ms; flip angle = 7°; 1 × 1mm in-plane resolution; slice thickness = 1mm, no gap; 188 slices).

The fMRI preprocessing and DC and RSFC networks calculation were all performed using the same procedures described above. The regions selected based on the results of the DC analyses in the HDT dataset were used as seed regions for seed-based RSFC analyses.

## Results

### PANAS

We calculated the scores of positive affect and negative affect of the PANAS before and after the HDT bed rest separately. Paired sample *T*-test showed that there were no significant differences for both the positive affect (*t* = −1.098, *p* = 0.289) and negative affect (*t* = 1.731, *p* = 0.104), which indicates that the participants did not have significant emotional changes after the HDT bed rest.

### Degree centrality

**Figure 1:**
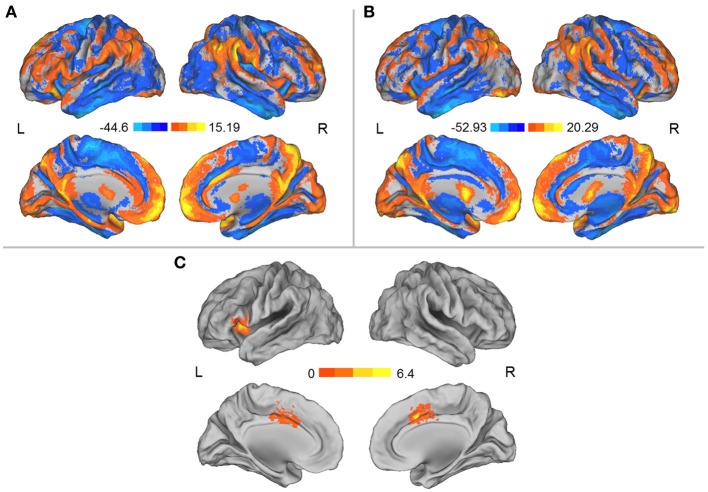
**(A)** Spatial distribution of the DCs before HDT bed rest. **(B)** Spatial distribution of the DCs after HDT bed rest. **(C)** Regions exhibiting significant changes in DC between the pre- and post-HDT bed rest conditions. The spatial distribution of the DCs was projected onto a surface brain using the Computerized Anatomical Reconstruction and Editing Toolkit (CARET) 5.62 (http://brainvis.wustl.edu/wiki/index.php/Caret:About).

Prior to the HDT bed rest, the spatial distribution of the weighted DC was highly localized in the posterior cingulate/ventral precuneus, occipital lobe, middle cingulate cortex (MCC), anterior cingulate cortex/medial prefrontal cortices, lateral prefrontal cortex, inferior parietal regions, insula, and thalamus (Figure 1A). This distribution is similar to that reported by Zuo et al. (2012). After HDT bed-rest, the spatial distribution of the weighted DC was also localized in the abovementioned regions, but the clusters were smaller (Figure 1B). Compared to the pre-HDT condition, significant decreases in the weighted DC were found in the left anterior insula (aINS) and MCC (BA24/32) after HDT bed rest (Figure 1C; Table 1). The findings obtained from the binarized graphs were highly similar to those obtained from the weighted graphs and thus are not presented.

**Table 1:** **Significant changes in degree centralities before and after HDT bed rest**.

**Cluster size**	**Hemisphere**	**Brain region**	**BA**	**MNI coordinates**	**Peak *T*-value**
**PRE > POST**					
106	Left	Insula	13/45/44	−39, 12, 9	6.40
88	bilateral	Middle cingulate cortex	24/32	9, 12, 36	5.39
**PRE < POST**					
None					

### RSFC networks

**Figure 2:**
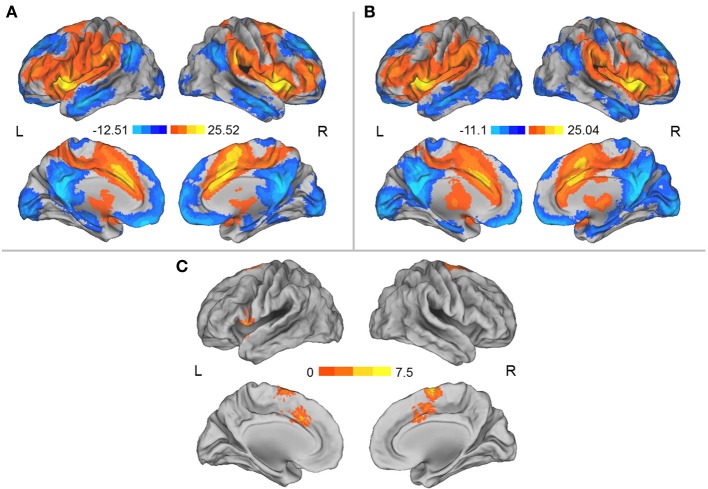
**(A)** Regions showing significant RSFCs with the left aINS before HDT bed rest. **(B)** Regions showing significant RSFCs with the left aINS after HDT bed rest. **(C)** Regions showing significant changes in positive RSFCs with the left aINS between pre- and post-HDT bed rest conditions. The images were created using CARET 5.62 (http://brainvis.wustl.edu/wiki/index.php/Caret:About).

**Figure 3:**
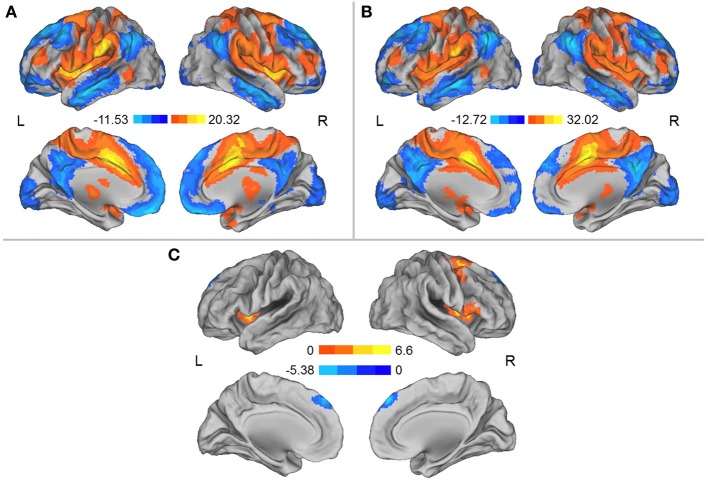
**(A)** Regions exhibiting significant RSFCs with the MCC before HDT bed rest. **(B)** Regions exhibiting significant RSFCs with the MCC after HDT bed rest. **(C)** Regions exhibiting significant changes in positive and negative RSFCs with the MCC between the pre- and post-HDT bed rest conditions. Warm colors represent the differences in the positive RSFCs, and cool colors represent the differences in the negative RSFCs. The images were created using CARET 5.62 (http://brainvis.wustl.edu/wiki/index.php/Caret:About).

We separately used each region in which the DC was influenced by simulated microgravity (i.e., the left aINS and the MCC) as a seed region for the mapping of the functional connectivity network. In general, these two regions exhibited similar patterns of functional connectivity prior to the HDT bed rest that included positive RSFCs with the bilateral insula, anterior and middle cingulate cortices, supplementary motor cortices (SMA), lateral prefrontal cortices and angular gyri and significant negative RSFCs with the bilateral medial prefrontal cortices, posterior cingulate cortices, superior frontal cortices, inferior temporal gyri, and inferior parietal gyri. After HDT bed rest, the spatial distributions of the RSFCs were also localized in the abovementioned regions, but the clusters were smaller (Figures 2A,B, 3A,B).

Compared to the results obtained prior to HDT bed rest, we found that the positive RSFCs between the left aINS seed region and the MCC and SMA and the positive RSFCs between the left aINS and its adjacent frontal cortex were significantly decreased after HDT bed rest (*p* < 0.05, corrected) (Figure 2C, Table 2). No significant increases in positive RSFCs or changes in negative RSFCs were found after HDT bed rest (*p* < 0.05, corrected).

**Table 2:** **Significant changes in functional connectivities before and after HDT bed rest using the left aINS as the seed region**.

**Cluster size**	**Hemisphere**	**Brain region**	**BA**	**MNI coordinates**	**Peak *T*-value**
**POSITIVE RSFCs: PRE > POST**					
139	Bilateral	Supplementary motor cortex	6	6, −6, 66	6.20
62	Left	Frontal operculum	13/44	−60, 9, 3	7.50
61	Bilateral	Middle cingulate cortex/supplementary motor area	24/32	−6, 18, 36	4.38

When the MCC was used as a seed region, we found that the positive RSFCs between this seed region and the left insula, the right insula and its adjacent inferior frontal gyrus and the right lateral superior frontal gyrus (SFG) and its adjacent precentral gyrus were significantly decreased after HDT bed rest (*p* < 0.05, corrected) (Figure 3C, Table 3). The negative RSFC between this seed region and the medial SFG was significantly decreased after HDT bed rest (*p* < 0.05, corrected) (Figure 3C, Table 3). No significant increases in positive RSFCs or negative RSFCs were found after HDT bed rest (*p* < 0.05, corrected).

**Table 3:** **Significant changes in functional connectivities before and after HDT bed rest using the MCC as the seed region**.

**Cluster size**	**Hemisphere**	**Brain region**	**BA**	**MNI coordinates**	**Peak *T*-value**
**POSITIVE RSFCs: PRE > POST**					
117	Right	Insula/inferior frontal gyrus	13/45/44	63, 18, 3	6.60
74	Right	Middle frontal gyrus/superior frontal gyrus	6	33, −3, 66	5.14
64	Left	Insula	13	−39, 6, 3	5.74
**NEGATIVE RSFCs: POST > PRE**					
119	Bilateral	Medial frontal gyrus/superior frontal gyrus	8	12, 51, 48	5.38

### Validation analyses

There were no significant differences in the weighted or binarized DC between the two scans for the validation dataset (*p* < 0.05, corrected). While using the left aINS or the MCC, in which the DC was influenced by simulated microgravity in the HDT dataset, as a seed region for seed-based RSFC analysis, there were no significant differences in functional connectivity strength between the two scans for each seed-based RSFC analysis.

## Discussion

Intrinsic functional connectivity provides a powerful and, at present, unique tool for the examination of the organization of the human brain (Buckner et al., 2013). A voxel-wise survey of the network centrality indices revealed decreased DCs in two regions, i.e., the left aINS and the anterior part of the MCC (also called the dorsal anterior cingulate cortex in many studies), in male volunteers after 45 days of −6° HDT bed rest. Furthermore, seed-based RSFC analyses revealed details about the altered functional networks that were related to these two regions after HDT bed rest. These disruptions in functional architecture indicated that a functional network anchored in the aINS and MCC was particularly influenced by simulated microgravity.

An emerging feature of brain architecture is that certain regions have strong connections with other regions within large-scale cortical networks and thus may play pivotal roles in the coordination of the flows of information that underlie various cognitive functions (Achard et al., 2006; Sporns et al., 2007; Hagmann et al., 2008; Buckner et al., 2009; Zuo et al., 2012). The intrinsic functional architecture of the brain has been found to change with development (Fair et al., 2007; Fransson et al., 2011) and aging (Tomasi and Volkow, 2012; Zuo et al., 2012), and its core regions are vulnerable to neuropsychiatric pathology (Buckner et al., 2009; Di Martino et al., 2013). The current findings indicate that simulated microgravity also alters this intrinsic functional architecture of the brain.

Some of the physiological effects of HDT bed rest may account for the changes in the aINS-MCC functional network that were observed after HDT bed rest. One of the direct effects of HDT bed rest is a shift in the distribution of fluid toward the upper body (Pavy-Le Traon et al., 2007; De La Torre et al., 2012). This cephalad fluid shift may alter the hemodynamics of the brain, and these alterations may include increases in cerebral blood flow (CBF), intracranial pressure, and oxygenated hemoglobin (for a review, please see Kawai et al., 2003). These hemodynamics changes that may occur during simulated microgravity may result in the observed changes in resting-state functional activities. This supposition is based on knowledge about the physiological meaning of the BOLD signal (Kim and Ogawa, 2012). Specifically, the coupling between the BOLD signal and CBF during rest is significant within the major regions of the resting-state networks (Liang et al., 2013; Tak et al., 2014), including the aINS-MCC network identified in the current study. However, these effects of upward shifts in body fluids on the hemodynamics of the brain are observed after short-term exposure to simulated microgravity (Kawai et al., 2003). Whether these effects remain after long-term exposure to simulated microgravity requires validation and the direct link between the effects of upward shifts in body fluids on the hemodynamics of the brain and the BOLD signal requires to be built.

Another critical effect of HDT bed rest or microgravity is the alteration of autonomic nervous function; this effect has been well documented after both short- and long-term HDT bed rest, and one manifestation of this effect is an alteration in the sympathetic–parasympathetic balance (Mano, 2005; Coupe et al., 2009). Autonomic function is regulated by a central autonomic network (Beissner et al., 2013). Some of the regions composing this network overlap with the regions identified in the current study. Specifically, the dorsal aINS is associated with parasympathetic regulation, and the bilateral MCC, SMA, and lateral prefrontal cortices and right ventral aINS are associated with sympathetic regulation (Beissner et al., 2013). Along this line, the decreases in the DCs in the aINS and MCC after HDT bed rest suggest decreased sympathetic and parasympathetic regulation. The decreased positive RSFCs related to these regions may reflect disruption of the balance between sympathetic and parasympathetic regulation.

From the network perspective, the observation of the coactivity of the aINS and MCC and their functional connectivity patterns provide critical insights for understanding the core functions of this network anchored in the aINS and MCC. The often-observed coactivation of the aINS and MCC across a variety of tasks suggests that these regions specifically respond to the degree of subjective salience, regardless of whether that salience is cognitive, homeostatic, or emotional (Craig, 2002, 2009; Critchley et al., 2004; Seeley et al., 2007). In a recent meta-analysis of human neuroimaging experiments that evaluated central autonomic processing, both the aINS and MCC were found to be part of a central autonomic network (Beissner et al., 2013). Studies of intrinsic functional connectivity have demonstrated the existence of an independent network that is anchored in the aINS and MCC (Dosenbach et al., 2007; Seeley et al., 2007; Taylor et al., 2009) and is distinct from the central executive network and the default mode network (Menon, 2011). Furthermore, the aINS is uniquely positioned to initiate control signals that enable switching between large-scale networks that are related to self-monitoring and task processing (Sridharan et al., 2008), and the MCC is thought to perform a similar function (Menon and Uddin, 2010). Therefore, these previous studies indicate that the aINS and MCC work as a network to detect the most relevant stimuli among internal and extrapersonal stimuli to facilitate appropriate behavioral responses, which would require changes in the sympathetic tone (Corbetta and Shulman, 2002; Seeley et al., 2007; Menon and Uddin, 2010; Legrain et al., 2011). Our findings suggest that the functions served by this aINS -MCC functional network were disrupted by simulated microgravity.

Additionally, we found that the MCC exhibited a decreased negative RSFC with the medial SFG. The medial SFG is a region in the default mode network whose functional correlates are typically ascribed to self-referential cognition (Buckner et al., 2008). The anti-correlations are often considered to serve a differentiating role in the segregation of neuronal processes that subserve opposite goals or competing representations (Fox et al., 2005). The decreased anti-correlation between the MCC and the medial SFG after HDT bed rest suggests that the competition between networks was abnormally decreased, which in turn suggests that the spontaneous activity of the default mode network may intrude during periods of active task-specific processing to producing periodic fluctuations in attention that compete with goal-directed activity. This default-mode interference hypothesis is thought to account for the cognitive dysfunction that occurs in psychiatric disorders (Sonuga-Barke and Castellanos, 2007). This decreased anti-correlation, combined with the decreased correlations within the aINS -MCC functional network, may contribute to the previously observed impairments in cognitive function that occur during microgravity (De La Torre et al., 2012).

Some caveats and unresolved issues must be discussed. We admit that it is difficult to reach firm conclusions regarding the specific functional roles of each region or network identified by resting-state fMRI because of the absence of a directly involved task. We have provided several explanations for our findings from the perspectives of changes in hemodynamics, autonomic dysfunction, and impairments in cognition that are based on current knowledge about the functional roles of the regions identified and the physiological effects of microgravity. However, there are other possibilities that may account for the current findings, such as emotional reactions associated with the feeling in the absence of gravity, decreases in vigilance due to the state of relaxation induced by the HDT position (Schneider et al., 2008), reductions in overall sensory stimulation and social interaction (Pavy-Le Traon et al., 2007) and orthostatic intolerance (i.e., the inability to maintain the upright position) following weightlessness (Furlan et al., 2009). The unchanged positive and negative affects measured by the PANAS suggest that these volunteers did not feel worse or better (relaxed) after HDT bed rest, at least at the scanning time point. This suggests that the changes in the aINS-MCC functional network after HDT bed rest may not result from the changes in the emotional state of these volunteers.

A limitation of the current study is the lack of a control group. This control group would have undergone two scans at the same time points as the experimental group but would not have participated in the HDT bed rest. It may be argued that the current findings are the result of a test-retest artifact. However, the high test-retest reliability of DC (Zuo et al., 2012) and RSFC (Yan et al., 2013b) measurements discount the possibility that the current findings are due to unstable BOLD signal changes between the scanning sessions. More importantly, we did not find significant differences in the weighted or binarized DC between two scans in another dataset. This dataset consists of 14 volunteers without being exposed to the bed rest procedure and the two MRI scans for each volunteers were performed with a comparable interval duration with the HDT dataset. While using the left aINS or the MCC, in which the DC was influenced by simulated microgravity in the HDT dataset, as a seed region for the seed-based RSFC analysis, we did not find significant differences in functional connectivity strength between the two scans for each seed-based RSFC analysis. These negative findings obtained in the validation dataset suggest that the current findings obtained in the HDT dataset less likely result from possible confounding factors related to scanning interval.

In conclusion, the current study found that simulated microgravity specifically influenced a functional network that was anchored in the aINS and MCC; we suggest that this network is involved in detecting biologically and cognitively relevant salient events to prioritize their access to attention and executive functions. The current findings might shed light on the neural bases of the physiological and psychological effects of long-duration spaceflights on astronauts. Furthermore, these findings may provide valuable insight into the neural mechanisms underlying the orthostatic intolerance that accompanies weightlessness and related human disorders, such as chronic orthostatic intolerance and baroreceptor failure (Furlan et al., 2009).

## Author contributions

All authors were involved in the design and implementation of the study and the writing of the manuscript. Authors Yuan Zhou, Xiao-Ping Chen, Chun-Hui Wang, Yan-Qiang Bai, Shan-Guang Chen, and Shu Li devised the concept and supervised the study. Author Yun Wang, Zhu-Yuan Liang, Cheng Tan, and Zhi-Qiang Tian collected the data. Authors Yuan Zhou, Yun Wang, and Dang Zheng carried out the analysis. Authors Yuan Zhou, Yun Wang, Li-Lin Rao, Xiao-Ping Chen, Chun-Hui Wang, Yan-Qiang Bai, Shan-Guang Chen, and Shu Li joined in the interpretation of data.

### Conflict of interest statement

The authors declare that the research was conducted in the absence of any commercial or financial relationships that could be construed as a potential conflict of interest.
